# Decoding the transcriptome from bulk RNA of infection-naïve versus imprinted patients with SARS-CoV-2 Omicron B.1.1.529

**DOI:** 10.1128/spectrum.02914-24

**Published:** 2025-07-09

**Authors:** Sissy Therese Sonnleitner, Samira Walder, Eva Hinterbichler, Ludwig Knabl, Roswitha Poernbacher, Gernot Walder

**Affiliations:** 1 Department of Virology, Medical Laboratory, Dr. Gernot Walder GmbH, Ausservillgraten, Austria; 2 Institute of Hygiene and Medical Microbiology, Medical University of Innsbruck 27280, Innsbruck, Austria; 3 Tyrolpath Obrist Brunhuber GmbH 703446 , Zams, Austria; Pontificia Universidad Catolica de Chile, Santiago, Chile

**Keywords:** transcriptome analysis, total RNA-seq, imprinted immune response, infection-naïve immune response, SARS-CoV-2 Omicron B.1.1.529, gene expression, vaccination

## Abstract

Total RNA-seq allows for the comprehensive detection of differentially expressed genes (DEGs), providing insight into immune responses during infection. In this study, we addressed the question of whether total RNA-seq can detect differences in gene expression and, therefore, immune responses between infection-naïve and imprinted patients. By sequencing all DEGs in the acute phase of a severe acute respiratory syndrome coronavirus 2 (SARS-CoV-2) Omicron B.1.1.529 infection, we examined the biological processes that characterize patients with different immune statuses. We differentiated between infection-naïve individuals (Group A), individuals imprinted by prior SARS-CoV-2 infection (Group B), and individuals first imprinted by vaccination (Group C). Infection status was confirmed by SARS-CoV-2-specific PCR; viral strain identification was performed via melting curve analysis; and immune status was assessed by chemiluminescent immunoassay. Our findings revealed significant transcriptomic differences between the study groups. Infection-naïve individuals exhibited the highest number of differentially expressed genes (1,526 DEGs), compared to 27 in previously infected individuals and only seven in vaccinated individuals. A key aspect of this difference was the expression of interferon-stimulated genes, which showed a progressive decline from infection-naïve to previously infected to vaccinated individuals. Notably, Group C displayed almost no upregulated genes but instead exhibited significant downregulation in pathways related to DNA metabolism. A key strength of our study is the inclusion of infection-naïve individuals at a time when most of the population had already been exposed to SARS-CoV-2. This rare opportunity allowed us to characterize the primary immune response to Omicron B.1.1.529 in infection-naïve individuals and compare it to immune responses shaped by prior infection or vaccination. The clear differences in gene expression between these groups highlight how prior exposure influences immune activation during acute infection.

**IMPORTANCE:**

Understanding how prior immune imprinting through infection or vaccination influences the transcriptomic response to severe acute respiratory syndrome coronavirus 2 (SARS-CoV-2) is crucial for optimizing vaccine strategies and predicting immune outcomes in future pandemics. A unique strength of our study is the inclusion of infection-naïve individuals at a time when most of the population had already been exposed to SARS-CoV-2. This rare opportunity allowed us to characterize the primary immune response to Omicron B.1.1.529 and compare it to responses shaped by prior infection or vaccination, providing valuable insights into how immune imprinting influences transcriptional activation during acute infection.

## INTRODUCTION

The comprehensive analysis of the transcriptome offers valuable insights into gene expression dynamics in response to varying physiological and pathological stimuli. Over the past decade, RNA sequencing (RNA-seq) has emerged as an indispensable tool in molecular biology, enabling high-resolution profiling of transcriptional activity and offering profound insights into cellular responses to infections and immune challenges (1–9). Unlike conventional gene expression methods, total RNA-seq allows for the detection of both coding and non-coding RNAs, offering a more comprehensive insight into gene regulatory networks (1–9).

The immune response to severe acute respiratory syndrome coronavirus 2 (SARS-CoV-2) infection varies significantly depending on prior immunological imprinting, whether through natural infection or vaccination. Previous studies have elucidated distinct transcriptional signatures differentiating infection-naïve individuals from those with pre-existing immunity due to prior exposure (10–14). However, the extent to which total RNA-seq can precisely delineate these immune responses at the transcriptomic level remains limited, as bulk RNA-seq provides an averaged view of gene expression across diverse cell populations, potentially obscuring heterogeneous immune responses occurring at the single-cell level. This limitation warrants further investigation, particularly in comparison to single-cell transcriptomics, which can resolve cell type-specific expression changes (15, 16).

In this study, we aimed to assess the utility of total RNA-seq in capturing differential gene expression (DGE) patterns between infection-naïve and immunologically imprinted patients during the acute phase of SARS-CoV-2 Omicron B.1.1.529 infection. Specifically, we sought to determine whether prior immune imprinting through infection or vaccination influences gene expression dynamics, particularly in pathways relevant to the antiviral response.

To address this, we analyzed the transcriptomes of infection-naïve, previously infected, and vaccine-imprinted individuals at two time points—during acute infection and convalescence—using total RNA-seq. We performed DGE and pathway enrichment analyses to identify transcriptional patterns associated with different immune imprinting backgrounds.

Our study provides key insights into how prior immune imprinting through infection or vaccination influences the transcriptional response to SARS-CoV-2. The most striking finding was that infection-naïve individuals exhibited a markedly stronger immune activation than both previously infected and vaccinated individuals, particularly in the expression of interferon-stimulated genes (ISGs). While previously infected and vaccinated individuals shared some transcriptional similarities, vaccinated individuals displayed a distinct transcriptional profile characterized by a pronounced downregulation of pathways related to DNA metabolism and cellular replication. These findings suggest that immune imprinting, particularly through vaccination, leads to an attenuated transcriptional response during acute infection with a reduced activation of innate immune pathways.

In summary, we were able to show that total RNA-seq is a robust and functional tool that can be used to examine and visualize transcription patterns at different stages of infection and compare different patient groups. Further studies would be desirable and beneficial to clarify the differences in gene expression patterns between vaccinated and unvaccinated study participants in larger patient cohorts. Ideally, the additional diagnostic tool of PBMC-seq (sequencing of RNA in peripheral blood mononuclear cells; PBMC) could provide deeper insights into the immunological pathways employed by individuals with different immunological statuses in their defense against infection (4, 17–19).

## MATERIALS AND METHODS

### Study population and recruitment

SARS-CoV-2 PCR-positive patients were recruited from volunteers diagnosed in the acute phase of infection between January and March 2022. All participants were infected with SARS-CoV-2 B.1.1.529, which was confirmed by specific PCR from nasopharyngeal swabs and subsequent melting curve analysis to determine the SARS-CoV-2 variant. The final study cohort consisted of 12 participants categorized into three groups based on immune status: infection-naïve (Group A), previously infected and recovered (Group B), and vaccinated (Group C). The serological assays confirmed the immunological classification, minimizing potential misclassification due to asymptomatic prior infections. Informed consent was obtained from each participant. Medical history, symptoms, and disease progression were recorded through structured interviews.

**Fig 1:**
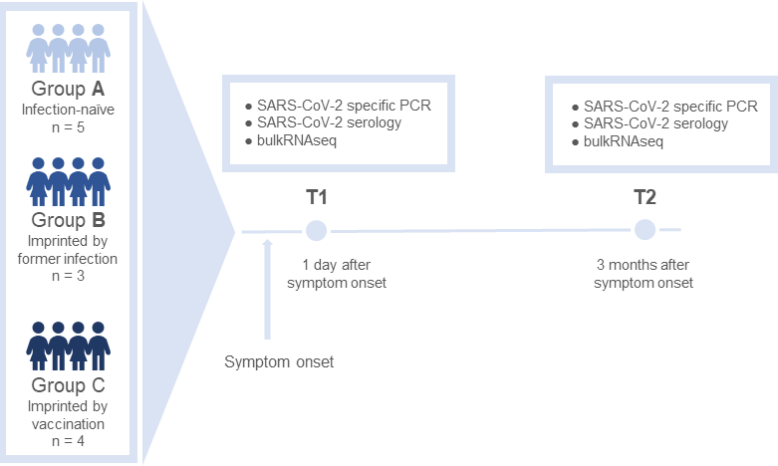
Schematic diagram of the study design. Participants were recruited among volunteers with PCR-confirmed infection with SARS-CoV-2 B.1.1.529. Samples were taken at time point 1 (T1) directly in the acute stage of the infection at the latest 1 day after SARS-CoV-2-positive PCR and at time point 2, 3 months after onset of symptoms, when the patient recovered from the infection with SARS-CoV-2. The participants were divided into three groups for further analysis: Group A—unvaccinated, infection-naïve patients; Group B—unvaccinated but recovered patients who already had a remembered infection with an earlier SARS-CoV-2 strain (imprinted by infection); and Group C—vaccinated patients (first imprinted by vaccination).

Samples were collected at two time points: T1, which occurred no later than 1 day after laboratory confirmation of a positive PCR test, and T2, 3 months later in the convalescent phase. Given that the incubation period for Omicron B.1.1.529 is estimated at 2–5 days (20), and considering the study’s recruitment strategy, we estimate that sample collection at T1 occurred approximately 3 to 7 days post-infection. At each time point, nasopharyngeal swabs were obtained to confirm acute infection, and blood samples were collected for serological titer determination via chemiluminescent immunoassay (CLIA) and transcriptome analysis using total RNA-seq. Total RNA was extracted from the leukocyte fraction of whole blood (buffy coat) after erythrocyte lysis. An overview of the study design is provided in Fig. 1.

Patients were, on average, 58.8 years old (SD = 15.2), with no significant differences in age between the groups. The group consisted of five men and seven women, whereby the distribution within the groups was homogeneous (Group A: 2/3; Group B: 2/1; Group C: 2/2). The severity of disease for each patient was assessed using a standardized questionnaire including age, gender, pre‐existing, as well as acute physical condition, and rated as mild, moderate, or severe course of disease according to definitions previously published (21). Briefly, in this study, we described a course as “mild” if the patient classified his symptoms as mild (e.g., fever, cough, sore throat, malaise, headache, muscle pain, nausea, vomiting, diarrhea, and loss of taste and smell) but did not experience shortness of breath or dyspnea, “moderate” if there were limitations in performing activities of daily living and/or subjective feelings of being ill and if the oxygen saturation measured by pulse oximetry (SpO2) was ≤94% on room air, and “severe” if the patient had to seek medical attention and/or needed to take medication to relieve symptoms and/or was bedridden and showed SpO2 of 30 breaths/min or lung infiltrates > 50%. The severity of the courses ranged from mild symptoms to moderate courses with respiratory distress and high fever and was evenly distributed throughout the three groups. A detailed list of the patients’ characteristics in terms of immune status, severity of SARS-CoV-2 infection, gender, and age can be found in Table S1 in the Appendix.

### CLIA SARS-CoV-2 TrimericS IgG

IgG antibodies reactive with the spike protein (S1/S2 domain) were determined using a commercially available CLIA (LIAISON SARS-CoV-2 TrimericS IgG). The assay was performed on the LIAISON XL analyzer according to the manufacturer’s instructions and yielded the binding antibody units per mL (BAU/mL) according to the World Health Organization’s international standards for the anti-SARS-CoV-2-immunoglobulin-binding activity (NIBSC 20-136).

### RNA extraction, PCR, and melting curve analysis

Peripheral blood was collected into EDTA-coated tubes (Vacuette, Greiner Bio-One, Austria). Five milliliters of well-mixed fresh whole blood was transferred into a sterile 50 mL tube and incubated with 15 mL of cell lysis solution (Promega, Germany) under sporadic and gentle inversion. After 10 min of incubation at room temperature, the tubes were centrifuged at 3,000 × *g* for 10 min. The supernatants were carefully removed and discarded without disturbing the visible white pellet. Any residual liquid at the bottom of the tube was collected after an additional spin and discarded. Two hundred microliters of chilled 1-thioglycerol/homogenization solution was added to the pellet, mixed by pipetting, and stored at −80°C until further RNA extraction.

RNA was isolated using the MagMAX-96 Total RNA Isolation Kit (Thermo Fisher Scientific, Waltham, MA, USA; Cat. no. AM1830). Briefly, 200 µL of the patient swab sample was mixed with 265 µL binding buffer, 5 µL proteinase K (20 mg/mL), and 5 µL extraction control (Thermo Fisher Scientific, Waltham, MA, USA) following the KingFisher extraction protocol for 200 µL sample volume. After incubation at room temperature for at least 15 min, samples were transferred into 96-well KingFisher deep well plates (Thermo Fisher Scientific, Waltham, MA, USA) containing 280 µL isopropanol and 2 µL Mag-Bind particles per well. RNA purification was performed using the KingFisher Flex Purification System (Cat. no. 5400620). RNA extraction was conducted from nasopharyngeal swabs for subsequent PCR analysis and from peripheral blood for RNA sequencing (RNA-seq).

Nasopharyngeal swabs were analyzed by RT-qPCR for the presence of SARS-CoV-2-specific RNA using the Simplexa Coronavirus Disease 2019 (COVID-19) Direct Kit (DiaSorin Molecular, Italy). Omicron strain identification was performed via melting curve analysis using primers targeting the SARS-CoV-2 Spike del69-70 deletion and the S371L and S373P mutations (TIB Molbiol GmbH, Germany) according to the manufacturer’s protocol. The results of the melting curve analysis were randomly verified by whole-genome sequencing using the Ion AmpliSeq SARS-CoV-2 Insight Research Assay (Cat. no. A51305, Thermo Fisher Scientific, USA) on an Ion Torrent S5 Plus System.

### Differential gene expression analysis

Raw data were processed on the Ion Torrent S5 Plus System and transferred to the Torrent server for mapping to the human reference genome hg19. Primary data analysis was performed using the AmpliSeq RNA plug-in with gene-level transcript quantification from sequence-read data and included quality control (QC) metrics. Quality criteria were defined according to the Thermo Fisher manual and set for this study as follows: read counts: >5 × 106; amplicon length: 120–130 bp. Raw data were normalized and analyzed using Transcriptome Analysis Console (TAC) software 4.0.2.15 (Thermo Fisher Scientific, USA) with the human reference genome hg19 to generate the differential gene expression (DEG) list. The DEG selection criteria were fold-change <-2 and >2, *P*-value < 0.05, and false discovery rate < 0.1. Gene ontology and pathway analyses were performed using Gene Set Enrichment Analysis (GSEA) (22) and Enrichr (23).

GSEA software compares the distribution of DEGs within the data to pre-defined ‘hallmark pathways’ (i.e., specific sets of genes that are characteristic of particular biological processes, signaling pathways, or functional themes). It calculates whether genes from a particular pathway or biological process are statistically significantly enriched compared to random genes. In the GSEA analysis, the FDR *q*-value (false discovery rate *q*-value) is used to correct for multiple testing. Enrichr integrates data from various resources, such as Gene Ontology, Kyoto Encyclopedia of Genes and Genomes (KEGG), WikiPathways, and others (10, 24, 25).

### Statistics

Dichotomous data were evaluated by a chi-squared test or Fisher’s exact test in the case of a small group size (*n*  <  60). A two-sided significance level of *P*  <  0.05 was used for determining statistical significance. After testing for distribution (Kolmogorov-Smirnov test), non-parametric continuous independent variables were compared using the Mann-Whitney *U* test for each time point. In the case of statistical testing of several groups of independent non-parametric data sets, the Kruskal-Wallis test was used. Dependent non-parametric variables were compared using the Wilcoxon rank test. All statistical analyses were performed with SPSS version 29.0 (Chicago, IL, USA). Additionally, a linear regression analysis was performed to examine the influence of age, sex, immune status, and disease severity on differential gene expression. The regression model was constructed using patient-based T1–T0 differences to account for intra-individual variation. Results indicated that none of the examined factors significantly influenced gene expression changes over time (*R*² = 0.162, *P* > 0.05). This analysis was included to further ensure the robustness of our statistical approach.

## RESULTS

### Characteristics of samples

In this study, we analyzed samples from 12 patients (five male, seven female) at two different time points—once at the time of acute infection (T1) with the SARS-CoV-2 Omicron variant confirmed by PCR, melting curve analysis, and symptomatology; and once at a second time point 3 months later when the patients had fully recovered and felt healthy; the symptoms had completely disappeared; and SARS-CoV-2 specific PCR was negative again (T2).

Five patients in our study made up the infection-naïve category, meeting the criteria of not being infected with SARS-CoV-2 until this laboratory-confirmed infection and unvaccinated. We confirmed this result serologically. The specific IgG titers averaged 3.5 BAU/mL (SD = 7.8), and only one of the five patients had an exceptionally low specific IgG titer of 17.5 BAU/mL; all others were completely negative.

Three patients were classified as imprinted by previous infection without vaccination in this study and assembled into Group B. These patients had a PCR-confirmed infection with SARS-CoV-2 other than strain Omicron B.1.1.529 in the previous year, which could be confirmed serologically. In this group, the mean specific IgG antibody titer was 41.2 BAU/mL (SD = 23.8).

Four patients in this study were classified in the comparative category “first imprinted by vaccination” or Group C. All of these patients had been vaccinated, and two had also experienced an earlier infection at least 12 months ago. Specific IgG antibody titers were at a mean level of 2,245.6 BAU/mL (SD = 1,448.5) in Group C. The type of vaccination was diverse: while one patient had triple Biontech vaccination, the other three patients in study group C had mixed events of imprinting—Astra and Biontech, Johnson and Biontech, and Biontech vaccination with records of breakthrough infections.

Significant differences in specific IgG AK titer were found between the naïve and vaccinated study groups (*P* = 0.006) but not between the naïve and recovered (*P* = 0.538) or recovered and vaccinated patients (*P* = 0.467; Kruskal-Wallis test). The patients were, on average, 58.8 years old (SD = 15.9). Detailed anonymized information on the patients can be found in Table S1 in the Appendix, and a brief overview of the characteristics of the grouping in this study is given in Table 1.

**TABLE 1:** Overview of the individual groups into which the patients were divided in this study and their characteristics regarding immune status in relation to SARS-CoV-2, sex, and age

	Status	No.	Male	[%]	Female	[%]
Group A	Infection-naïve	5	2	40.0	3	60.0
Group B	First imprinted by previous infection	3	2	66.7	1	33.3
Group C	First imprinted by vaccination	4	2	50.0	2	50.0
Total		12	6	50.0	6	50.0

The SARS-CoV-2 variants responsible for the acute-phase infections in this study were SARS-CoV-2 Omicron BA.1 and BA.5, as confirmed by melting curve analysis and random confirmation by whole genome sequencing.

There were no significant differences in the viral loads of the nasopharyngeal swabs represented by the Ct values in the SARS-CoV-2-specific PCR. In Group A, the infection-naïve patient group, and Group C, the study group of patients imprinted by vaccination, the mean Ct values were 29 and 27 (SD = 7.0 and 5.0), respectively, corresponding to viral loads of approximately 68 and 394 copies/mL. The viral loads of Group B (imprinted by previous SARS-CoV-2 infection) were insignificantly lower with a mean Ct of 34 (SD = 2) and a mean viral load of 0.8 copies/mL. Although viral loads appeared lower in Group B, the differences were not statistically significant (*P* > 0.05). Given the small sample size, it is important to acknowledge that this may limit the ability to detect significant differences (*P* = 1.0, 0.2, 0.28; Kruskal-Wallis test).

### Identification of DEGs

A total of 20,809 genes were analyzed using TAC software 4.0.2.15 (Thermo Fisher Scientific). Normalization was performed with the AmpliSeq RNA plug-in, which enabled gene-level transcript quantification and included QC metrics, such as read depth, mapping quality, and transcript coverage, to ensure data reliability. Patients were grouped into A (infection-naïve), B (imprinted by previous infection), and C (first imprinted by vaccination).

Group A exhibited the highest number of DEGs (1,526), while Group B had 27 DEGs, and Group C only seven DEGs during the acute phase (T1) compared to the convalescent phase (T2). The differences in DEG levels between groups were highly significant (A:B *χ*² = 580.6; A:C *χ*² = 1,422; B:C *χ*² = 41; *χ* test for normally distributed Ct values). Significant differences in DEGs were observed among the three groups.

A combined analysis across all groups (ABC) identified 128 upregulated and one downregulated DEG. To further investigate potential factors influencing differential gene expression, we conducted a linear regression analysis incorporating age, sex, immune status, and disease severity as covariates. The model explained only 16.2% of the variability in gene expression changes (*R*² = 0.162). Sex was statistically significant (*β* ≈ 0, *P* < 0.001), but the effect size was negligible. No significant associations were found for age, immune status, or disease severity (*P* > 0.05). This suggests that the observed DEG differences were primarily driven by immune imprinting rather than these covariates. A complete list of up- and downregulated genes per group is provided in Table 2.

**TABLE 2:** Number of up- and downregulated genes in the individual patient groups

	A	[%]	B	[%]	C	[%]	ABC total	[%]
Upregulated	1,526	99.7	27	54.0	7	7.1	128	99.2
Downregulated	5	0.3	23	46.0	91	92.9	1	0.8
Total	1,531	100.0	50	100.0	98	100.0	129	100.0

**Fig 2:**
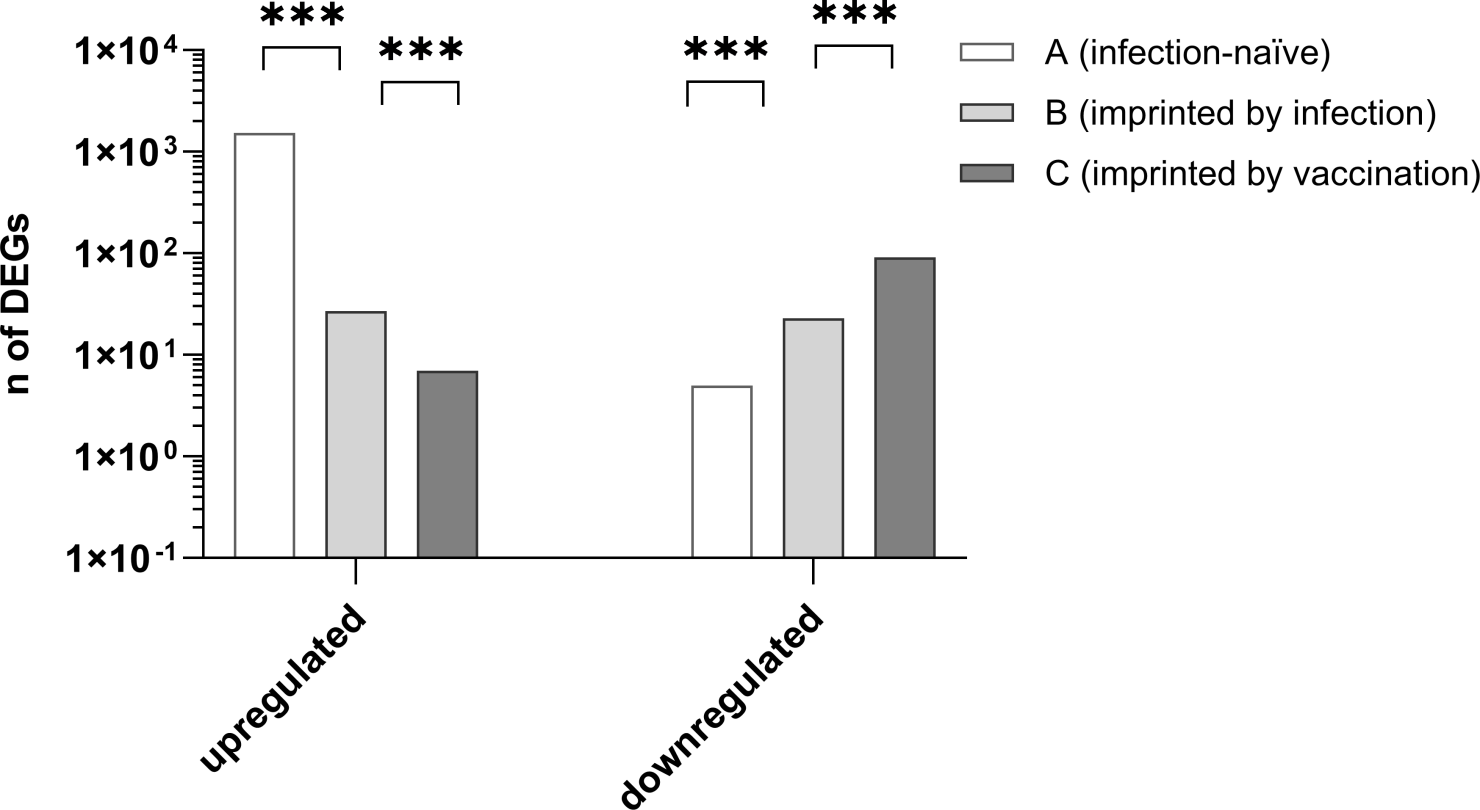
Comparison of the numbers of up- and downregulated differentially expressed genes (DEGs) in the three different study groups of infection-naive (Group A), previous SARS-CoV-2 infection-impaired (Group B), and vaccination-impaired (Group C) patients in the acute stage of infection with Omicron B.1.1.529 compared to the recovered status of the patients 3 months later. The number of upregulated genes was significantly higher in the infection-naive group A (*P* > 5 × 10^−4^). ***Statistical significance between groups (*P* < 0.001).

All three groups differed significantly in the number of up- and downregulated genes. Group A had the highest number of upregulated DEGs (*P* < 5 × 10^−^⁴), as shown in Fig. 2.

**Fig 3:**
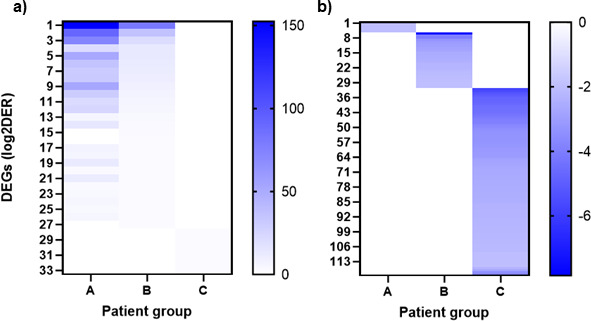
Heatmaps comparing the number of up- and downregulated differentially expressed genes (DEGs) in the three patient groups, A (infection-naïve), B (imprinted by previous infection), and C (first imprinted by vaccination), during acute infection with SARS-CoV-2 Omicron (B.1.1.529). Each row corresponds to a gene, each column to a sample. The colors represent expression: high levels of expression are shown in intense blue, while weaker levels fade to white. Patterns of genes with similar expression profiles or clusters become visible. In the heatmap of upregulated DEGs, there appears to be an overlap between groups A and B, although B shows significantly weaker signal strength. (a) Heatmap of upregulated DEGs (log2 fold change > 2, *P* < 0.05, FDR < 0.1). (b) Heatmap of downregulated DEGs (log2 fold change < -2, *P* < 0.05, FDR < 0.1).

In Group A, a total of 1,531 genes were differentially expressed in the acute phase of infection (T1) compared to the convalescent phase 3 months later (T2) with a fold change > 4 corresponding to a log2 fold change (Log2FC) > 2 based on the TAC differential expression analysis. Of these 1,531 DEGs, 1,526 were upregulated, and five were downregulated (99.7% versus 0.3%). In Group B (unvaccinated and recovered), only 50 DEGs were detectable with a Log2FC > 2, of which 27 DEGs (54%) were upregulated, and 23 (46%) downregulated. In Group C (first imprinted by vaccination), 98 DEGs were found with a Log2FC > 2, of which only seven (7.1%) were upregulated, and 91 (92.9%) were downregulated. Fig. 3 presents heatmaps illustrating that gene expression patterns are largely group-specific, with a minimal overlap between the three groups.

### Expression of interferon-stimulated genes

Since ISGs play a key role in antiviral immune responses and are often among the earliest markers of immune activation, we next examined their expression across the three study groups. Differences in the ISG expression may provide insights into how prior infection or vaccination modulates immune responses to SARS-CoV-2. A focused analysis of interferon-stimulated genes, including IFI6, IFIT1, IFITM3, and MX1, revealed a significant variation in expression levels between the three study groups. At the acute phase of infection (T1), the expression of ISGs was highest in Group A, followed by Group B, and lowest in Group C. The mean log2-transformed expression values in Group A were 10.19, significantly higher than in groups B (8.91) and C (7.33). A one-way analysis of variance confirmed highly significant differences between the groups (*P* = 4.27 × 10⁻⁵).

Post-hoc Tukey-HSD tests demonstrated that all pairwise comparisons were statistically significant. The difference in the ISG expression between groups A and B was 1.28 log2-fold (*P* = 0.027), while the contrast between groups A and C was even more pronounced with a 2.86 log2-fold difference (*P* < 0.001). Additionally, Group B exhibited a significantly higher ISG expression than Group C (1.58 log2-fold, *P* = 0.011).

### GSEA analysis of DEGs

The top 500 up- and downregulated DEGs in each group were entered into GSEA, and overlap in the hallmark gene sets was calculated as a first step. To improve the robustness of the analysis, the FDR *q*-value was set to<0.01 in our analysis.

Due to the low number of upregulated genes in Group C (first imprinted by vaccination; *n* of upregulated genes = 7), the GSEA analysis was not possible due to insufficient statistical power. Therefore, the GSEA analysis of upregulated genes was restricted to the data of Group A (infection-naïve) versus Group B (imprinted by previous SARS-CoV-2 infection). However, in Group C, the analysis of downregulated genes was possible due to the high number (*n* = 98 downregulated DEGs).

**Fig 4:**
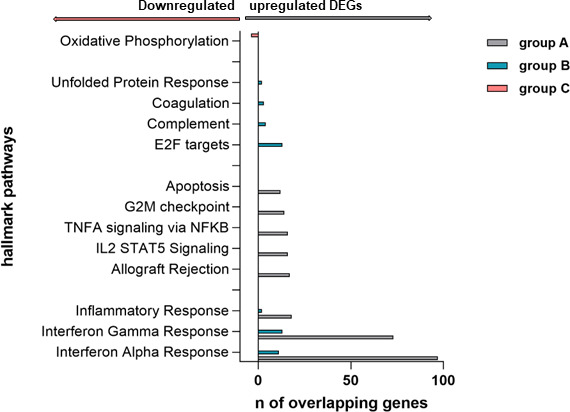
Inclusion of the 500 most up- and downregulated genes in GSEA revealed overlapping genes in different hallmark pathways between groups A and B. While DEGs with a high statistical probability were found in both groups that were associated with the biological processes of inflammatory response, interferon-alpha, and gamma response, only in Group A were DEGs found to be associated with apoptosis, G2M checkpoint, TNF-alpha signaling via NFKB, IL2-STAT5 signaling, and allograft rejection. Only in Group B did we find DEGs involved in the biological processes of unfolded protein response, coagulation, complement, and E2F targets. Only those overlapping genes in hallmark pathways with a statistical probability of *P* > 0.5 × 10^−3^ were shown.

The number of overlapping genes ranged from 97 (overlapping with the characteristic interferon-alpha response in Group A) to two (characteristic unfolded protein response in Group B). Both groups A and B had upregulated DEGs that showed a significant overlap with the characteristic pathways “inflammatory response,” “interferon gamma response,” and “interferon alpha response.” However, the DEGs differed between the two groups in the hallmarks that showed gene patterns for “unfolded protein response," “coagulation," “complement,” and “E2F targets.” DEGs with a significant overlap in these hallmark pathways were mainly expressed by patients in Group B (imprinted by previous infection). An overview of the number of overlapping genes in the different study groups with DEGs associated with different hallmark pathways and their listing can be found in Fig. 4.

**Fig 5:**
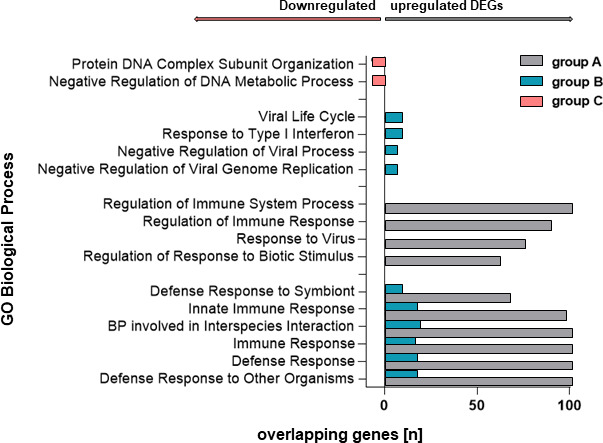
Inclusion of the top 500 up- and downregulated genes in GSEA revealed overlapping genes in different biological process (BP) gene ontologies (GO) between groups A, B, and C. While groups A (infection-naïve) and B (imprinted by previous infection) showed some overlapping genes in the same biological processes (defense response to symbionts, innate immune system), this study also found biological processes exclusive to a single group. In the GO:BP analysis, the 91 genes downregulated in the acute phase of infection with SARS-CoV-2 B.1.1.529 in Group C (imprinted by vaccination) were mainly associated with the protein-DNA complex subunit organization (7/91) or the negative regulation of DNA metabolic processes (6/91).

In a further GSEA analysis, the 500 most and most highly up- or downregulated DEGs were examined for overlapping events within the biological process gene ontology (GO:BP) and visualized in Fig. 5. Again, 500 upregulated genes were analyzed for Group A, 27 upregulated genes for Group B, and 91 downregulated genes for Group C. For Group A, the calculation of DEGs in the context of GO:BP showed that the upregulated processes mainly included the regulation of immune system processes (105/500) and response (81/500), response to a virus (75/500), as well as to a biotic stimulus (61/500). Meanwhile, the BPs for Group B mainly included viral life cycle (9/27), response to type I interferon (7/27), and negative regulation of viral processes and viral genome replication (7 and 6/27, respectively). Some of the 91 downregulated genes in the acute phase of SARS-CoV-2 B.1.1.529 infection in study group C patients were primarily associated with the protein-DNA complex subunit organization (7/91) or the negative regulation of DNA metabolic processes (6/91).

### Enrichr analysis

Further analysis of up- and downregulated DEGs with LOG2FC > 2 between the acute (T1) and convalescent (T2) phases of SARS-CoV-2 B.1.1.529 infection in patients with different immune statuses was performed using another web-based gene set enrichment analysis tool, Enrichr software (23). Enrichr integrates data from different resources, such as Gene Ontology, KEGG, WikiPathways, and others (10, 24, 25). For this purpose, the total number of up- or downregulated DEGs for each group was entered into the database (not only the first 500 up- or downregulated genes as in the GSEA database), and the results of the WikiPathways analysis were compared. A combined score was calculated from the adjusted *P*-value and odds ratio to assess the strength of the association between the accumulation of DEGs and the pathway, thus representing the robustness of the result. Table 3 shows the WikiPathways calculated using the Enrichr analysis, as well as the odds ratio and the combined scores as measures of the statistical probability of a true correlation between the clustering of DEGs and the named pathways. The combined scores in our data set ranged from 55.39 (immune response to tuberculosis; Group A) to 3,428.37 (complement activation; Group B) and were considered robust if they were greater than 300. Data were also processed using Appyter software (https://appyters.maayanlab.cloud/). Appyter is a web-based platform connected to the Enrichr platform and an interactive tool that enables data-driven analysis workflows, data integration, and visualization without extensive programming skills.

**TABLE 3:** Overview of the WikiPathways identified in the Enrichr analysis^*a*^

Group A	WikiPathway	WP no.	Adjusted *P-*value	Odds ratio	Combined score
	Type I interferon induction and signaling during SARS-CoV-2 infection	WP4868	8.38E−06	10.05	180.19
	DDX1 as a regulatory component of the Drosha microprocessor	WP2942	2.68E−02	16.18	112.11
	Homologous recombination	WP186	8.75E−03	10.41	88.2
	Pathways of nucleic acid metabolism and innate immune sensing	WP4705	4.68E−03	9.45	87.89
	Host-pathogen interaction of human coronaviruses—interferon induction	WP4880	1.68E−03	6.09	65.96
	Simplified depiction of MYD88 distinct input-output pathway	WP3877	8.75E−03	7.73	65.04
	Biomarkers for pyrimidine metabolism disorders	WP4584	1.71E−02	8.10	61
	IL-10 anti-inflammatory signaling pathway	WP4495	3.29E−02	8.67	57.69
	15q11.2 copy number variation syndrome	WP4940	4.96E−02	9.71	56.03
	Immune response to tuberculosis	WP4197	8.75E−03	6.49	55.39

Group B	WikiPathway	WP no.	Adjusted *P*-value	Odds ratio	Combined score
	Complement activation	WP545	3.50E−07	192.8	3,428.37
	Complement and coagulation cascades	WP558	3.50E−07	85.42	1,549.67
	Oxidative damage	WP3941	2.87E−06	96.31	1,471.02
	Host-pathogen interaction of human coronaviruses—MAPK signaling	WP4877	1.13E−04	75.53	837.92
	Microglia pathogen phagocytosis pathway	WP3937	1.29E−04	67.35	725.55
	Immune response to tuberculosis	WP4197	2.02E−03	76.01	588.08
	Allograft rejection	WP2328	5.50E−05	40.69	489.64
	Non-genomic actions of 1,25 dihydroxyvitamin D3	WP4341	6.24E−04	36.59	330.97
	Type II interferon signaling (IFNG)	WP619	4.67E−03	45.57	309.02
	COVID-19 adverse outcome pathway	WP4891	4.64E−02	54.83	214.32

Group C	WikiPathway	WP no.	Adjusted *P*-value	Odds ratio	Combined score
	Cholesterol biosynthesis pathway	WP197	1.78E−01	34.39	212.57

^*a*^ The name of the pathway, WikiPathway identification number (WP no.), adjusted *P*-value, and the odds ratio are listed.

The table lists the pathway name, WikiPathway identification number (WP no.), adjusted *P*-value, and odds ratio. The latter two parameters contribute to the combined score, which serves as a reliability measure. The highest combined scores are observed in the top three pathways of Group B, indicating a strong statistical association between the accumulation of DEGs and the corresponding biological processes.

**Fig 6:**
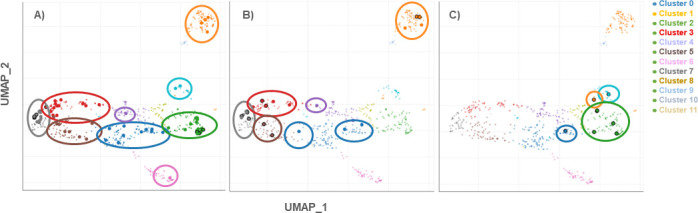
Scatter plots of all terms in the WikiPathway_2021_Human gene set library. The scatterplot is organized so that similar gene sets are clustered together. Clusters are computed using the Leiden algorithm on the Appyter platform (https://appyters.maayanlab.cloud/). Points are plotted on the first two UMAP dimensions. Larger points outlined in black represent significantly enriched terms. The comparison of the three scatter plots of groups A, B, and C gives a good indication of the extent to which genes are overexpressed at the time of acute infection with SARS-CoV-2 B.1.1.529 and into which gene clusters they would be classified according to the WikiPathways analysis. The differently colored clusters can be grouped into the following overarching themes: • **Cluster 0**—cellular signaling and regulatory pathways **• Cluster 1**—metabolism and cellular response pathways **• Cluster 2**—cellular metabolism and maintenance pathways **• Cluster 3**—immune response, cell signaling, and disease pathways **• Cluster 4**—cellular regulation and disease mechanisms **• Cluster 5**—cellular signaling and immune response pathways **• Cluster 6**—neurological and metabolic disorders pathways **• Cluster 7**—innate immune response and host-pathogen interactions **• Cluster 8**—developmental and genetic pathways **• Cluster 9**—metabolism and cell differentiation pathways **• Cluster 10**—metabolism and disease-related pathways **• Cluster 11**—mitochondrial function, energy metabolism, and cellular health pathways.

Using the Appyter platform, the pathways identified in Enrichr can be mapped to specific gene clusters, as shown in Fig. 6. The 27 upregulated DEGs of Group B (imprinted by previous SARS-CoV-2 infection) were found in seven out of 11 clusters in the WikiPathway analysis, namely, in clusters 0, 1, 2, 3, 4, 5, and 6. Clusters 2 (cellular metabolism and maintenance pathways) and 9 (metabolism and cell differentiation pathways) were less pronounced in this group compared to Group A.

In Group C (first imprinted by vaccination), seven upregulated and 91 downregulated DEGs were found in the acute phase of infection with SARS-CoV-2 B.1.1.529. Analysis of the downregulated genes revealed that they belonged to four of the 11 WikiPathway clusters, namely, clusters 0, 1, 2, and 9 (i.e., clusters likely involved in the biological processes: cellular signaling and regulatory pathways (Cluster 0), metabolism and cellular response pathways (Cluster 1), cellular metabolism and cellular response pathways (Cluster 2), and cellular metabolism (Cluster 3). Using Appyter, the DEGs in our study are plotted in a formation where similar gene sets are clustered together and visualized in scatter plots in Fig. 6, giving us an overview of the differences between patient groups A (infectious-naïve), B (imprinted by previous infection), and C (first imprinted by vaccination).

### Venn diagram in TAC

TAC software provides the ability to analyze differences in gene expression patterns in different study groups by comparing overlapping DEGs in a Venn diagram.

**Fig 7:**
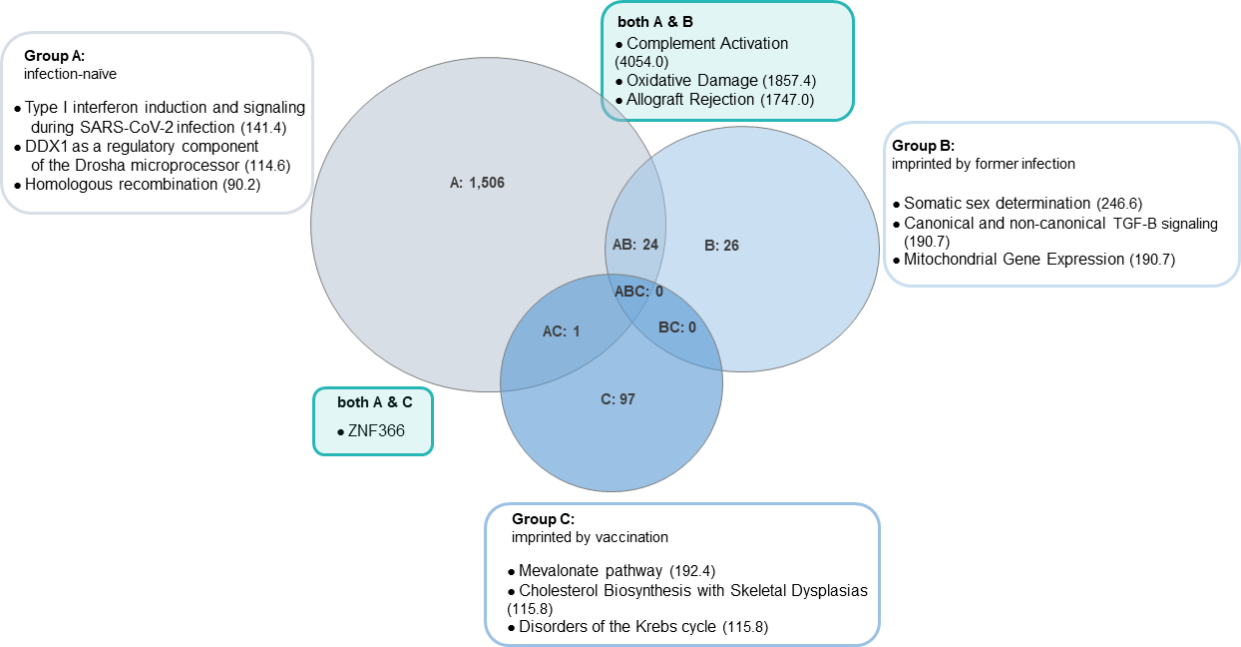
Venn diagram to visualize the relationship of up- and down-regulated DEGs between the different study groups. The number of overlapping DEGs with WikiPathways is shown in the respective circles of the diagram. There are overlapping DEGs between groups A and B (*n* = 24) and one between A and C (ZNF366; Zinc Finger Protein 366) but none between B and C. The WikiPathways with the highest combined scores are shown, as well as the location of the most upregulated genes in the gene cluster. The combined score is given in brackets, which, as a combination of *P*-value and odds ratio, gives a good indication of the robustness and reliability of the statement. In this study, we considered combined scores > 300 to be robust. Again, the differently colored clusters can be summarized into the following overarching topics: **• Cluster 0**—cellular signaling and regulatory pathways **• Cluster 1**—metabolism and cellular response pathways **• Cluster 2**—cellular metabolism and maintenance pathways **• Cluster 3**—immune response, cell signaling, and disease pathways **• Cluster 4**—cellular regulation and disease mechanisms **• Cluster 5**—cellular signaling and immune response pathways **• Cluster 6**—neurological and metabolic disorders pathways **• Cluster 7**—innate immune response and host-pathogen interactions **• Cluster 8**—developmental and genetic pathways **• Cluster 9**—metabolism and cell differentiation pathways **• Cluster 10**—metabolism and disease-related pathways **• Cluster 11**—mitochondrial function, energy metabolism, and cellular health pathways.

The Venn diagram in Fig. 7 shows whether and to what extent the DEGs in each group overlap. The analysis included both up- and downregulated DEGs with a LOG2FC > 2. The overlap between groups A and B was 24 genes; that between B and C was zero; and that between A and C was one gene. No DEGs were shared by all three groups.

In the next step, we investigated which biological processes and functions lie behind the individual DEGs that are (i) shared between groups or (ii) only occur in individual groups. The WikiPathways and biological functions to which the DEGs are assigned are not equally robust, as represented by the combined score—a combination of *P*-value and odds ratio. In our study, results in the Enrichr analysis with combined scores < 300 should be treated with caution. Thus, the clear and statistically robust results are those of the cluster analysis of shared DEGs between groups A (infection-naïve) and B (imprinted by previous infection). The 24 DEGs shared by these two groups have the highest concordance with the gene expression patterns associated with the pathways of complement activation (combined score [cs] 4,054.0), oxidative damage (cs 1,857.0), and allograft rejection (cs 1,747.0). These 24 DEGs shared by groups A and B cluster in the scatter plot mainly with genes from clusters 0, 1, 3, 4, 5, and 7 (cellular signaling and regulatory pathways, metabolism and cellular response pathways, immune response, cell signaling and disease pathways, cellular regulation and disease mechanisms, cellular signaling and immune response pathways, and innate immune response and host-pathogen interactions, respectively).

A listing of all WikiPathways specific to each individual group, as well as split between groups, including adjusted *P*-value and combined score, can be found in the Appendix section (Table S2).

## DISCUSSION

In this study, we used transcriptome analysis to investigate differences in gene expression between the acute and convalescent phases of SARS-CoV-2 Omicron B.1.1.529 infection across three study groups: infection-naïve individuals, individuals with prior infection (infection-imprinted), and individuals with prior vaccination (vaccine-imprinted).

Our findings reveal significant transcriptomic differences between the acute and convalescent phases, with infection-naïve individuals exhibiting the highest number of DEGs during the acute phase and 1,526 upregulated DEGs compared to 27 in previously infected individuals and seven in vaccinated individuals. This suggests a strong innate immune activation in naïve individuals, whereas prior antigenic exposure through infection or vaccination appears to reduce the necessity for widespread transcriptional changes. The GSEA and Enrichr analysis confirmed that these DEGs were primarily associated with immune activation, including interferon responses and inflammatory pathways, indicating that the transcriptomic changes were largely infection-specific. Individuals with prior infection (Group B) showed a more attenuated response, suggesting partial immune imprinting, while vaccinated individuals (Group C) exhibited the lowest DEG counts, primarily reflecting metabolic downregulation rather than an active immune response.

A particularly pronounced difference was observed in the expression of ISGs, which showed a progressive decline from infection-naïve to previously infected to vaccinated individuals. ISG expression, particularly for IFI6, IFIT1, IFITM3, and MX1, was highest in naïve individuals, lower in previously infected individuals, and lowest in vaccinated individuals (log2 values: 10.19 > 8.91 > 7.33, *P* = 4.27 × 10⁵).

These findings show that the ISG expression was lower in previously infected and vaccinated individuals compared to infection-naïve individuals during acute Omicron infection. The progressively lower ISG expression from infection-naïve to previously infected to vaccinated individuals suggests a dampened interferon response in groups B and C relative to Group A.

The robust immune activation observed in infection-naïve individuals is consistent with previous studies, which demonstrate that primary SARS-CoV-2 exposure induces pronounced transcriptomic alterations, particularly in pathways involved in DNA repair, oxidative stress, and inflammation (11–14). This response appears to be broader and more intense than the transcriptional activation observed in imprinted individuals, further supporting the concept of immune imprinting (16, 18).

To further evaluate potential confounding variables, we performed a regression analysis incorporating age, sex, immune status, and disease severity as covariates. Our findings confirmed that none of these factors, except for sex, significantly influenced gene expression changes. Although sex was statistically significant (*P* < 0.001), the effect size was small (*η*² = 0.013, Cohen’s *d* = 0.23), indicating that while a measurable association exists, it is unlikely to be biologically relevant.

Further analysis revealed that while groups A and B shared activation of interferon-alpha (IFN-α) and interferon-gamma (IFN-γ) responses, Group A exhibited a broader range of immune activation, including TNF-α signaling, apoptosis, and IL2-STAT5 pathways. These findings suggest that the initial immune response to SARS-CoV-2 in naïve individuals is more extensive, while prior exposure through infection or vaccination leads to a more restrained transcriptional response. Notably, vaccinated individuals (Group C) demonstrated a distinct transcriptomic signature dominated by metabolic downregulation rather than classic immune activation. This aligns with previous studies indicating that vaccine-induced immunity leads to more targeted and efficient immune responses compared to natural infection (26–28).

In this study, we observed that infection-naïve individuals exhibited a pronounced upregulation of ISGs during the acute phase of SARS-CoV-2 Omicron B.1.1.529 infection. This robust ISG response aligns with previous research indicating that initial SARS-CoV-2 exposure triggers significant interferon production and ISG activation, which are critical components of the innate immune response against viral infections (29).

Conversely, individuals with prior SARS-CoV-2 infection or vaccination demonstrated a more attenuated ISG response during acute infection. This attenuation may be attributed to immune imprinting, where previous antigenic exposure modulates subsequent immune responses, leading to a more controlled and efficient activation upon re-exposure.

These findings suggest that while the innate immune system mounts a vigorous response during primary SARS-CoV-2 infection, prior immunological exposure through infection or vaccination can modulate this response, potentially reducing the risk of overactivation and associated immunopathology. Understanding the dynamics of ISG expression in different immunological contexts is essential for developing targeted therapeutic strategies and optimizing vaccine designs to elicit balanced and protective immune responses (30).

Moreover, genetic variations in the JAK-STAT signaling pathway may also contribute to the observed differences in immune responses upon SARS-CoV-2 infection. The JAK-STAT pathway plays a central role in mediating antiviral immunity and cytokine signaling, and its dysregulation has been implicated in severe COVID-19 cases due to an excessive inflammatory response. Notably, mutations affecting the components of this pathway can influence the intensity and duration of immune activation, potentially accounting for individual variability in transcriptomic responses to infection (31). Future studies incorporating host genomic analyses alongside transcriptomic profiling could provide deeper insights into the genetic determinants of immune imprinting in SARS-CoV-2 infection.

Our results contrast with those of previous transcriptome studies, which reported stronger transcriptomic activation in vaccinated individuals, particularly in pathways related to interferon responses and cytokine signaling (19, 32). However, these studies focused on recently vaccinated individuals, whereas our cohort had a longer time gap between vaccination and infection, likely influencing the observed differences. Previous studies investigating vaccinated individuals often observed stronger transcriptomic activation, particularly in pathways associated with IFN-α/γ, complement activation, and IL-6/JAK-STAT signaling. However, these studies typically included participants with more recent vaccination (<3 months before infection), whereas in our cohort, vaccination occurred at least 12 months prior to infection. This suggests that the timing of immune imprinting plays a crucial role in transcriptomic responses, emphasizing the need for longitudinal studies to capture the full spectrum of immune memory effects.

Our findings also underscore the challenges of using bulk RNA sequencing to fully resolve the complexity of immune responses. While strong transcriptomic activation was detected in naïve individuals, the expected T- and B-cell responses in vaccinated individuals were not prominently represented in our data set. This may be due to the inherent limitations of bulk RNA-seq in distinguishing cell type-specific transcriptional changes but could also reflect the analytical tools available at the time of this study. Future studies integrating single-cell RNA sequencing (scRNA-seq) or PBMC-targeted RNA-seq, as well as expanded bioinformatics approaches, may provide a more detailed characterization of these immune mechanisms.

This study has several limitations. Most notably, the small sample size limits statistical power, particularly in pathway enrichment analyses. Second, transcriptome profiling was conducted at only two time points—during acute infection and 3 months post-infection—potentially missing transient immune activation patterns. Third, bulk RNA-seq captures gene expression across all circulating cells, limiting its ability to resolve cell type-specific transcriptional changes. More targeted approaches, such as PBMC-seq, could provide a greater resolution of immune cell-specific responses. Furthermore, our data set did not prominently capture the expected T- and B-cell responses in vaccinated individuals. This may reflect the limitations of bulk RNA-seq in detecting adaptive immune responses or constraints in the analytical tools available at the time of this study. Future research should incorporate larger cohorts and longitudinal sampling to track immune response dynamics more comprehensively. Additionally, integrating single-cell transcriptomics or immune cell-specific RNA-seq, along with expanded bioinformatics approaches, could further elucidate the immunological mechanisms underlying the observed differences, particularly the metabolic downregulation in vaccinated individuals.

Despite these limitations, our study provides important insights into how prior immune imprinting shapes the transcriptional response to SARS-CoV-2 infection. This rare opportunity allowed us to analyze a true primary immune response to SARS-CoV-2 Omicron B.1.1.529 and compare it with imprinted responses shaped by prior exposure.

### Conclusion

This study provides key insights into how prior immune imprinting through infection or vaccination influences the transcriptional response during acute SARS-CoV-2 infection. Our findings confirm that infection-naïve individuals exhibit a markedly stronger immune activation than both previously infected and vaccinated individuals, particularly in the expression of ISGs.

One of the most striking findings was the significant difference in DEGs between infection-naïve and immunologically imprinted individuals. The robust transcriptional activation observed in naïve individuals suggests a markedly different innate immune response compared to previously infected or vaccinated individuals. These differences are well-supported, but our ability to further dissect their underlying mechanisms is limited by the resolution of bulk RNA sequencing and the bioinformatics strategies implemented in this study.

Importantly, this attenuated response did not correlate with negative clinical outcomes. On the contrary, individuals with prior immune imprinting exhibited insignificantly shorter infectious episodes, as indicated by slightly higher Ct values in nasopharyngeal swabs and a less prolonged viral presence in imprinted groups. These findings provide further evidence that immune imprinting in SARS-CoV-2 infection attenuates the transcriptional response without compromising the ability to control viral replication.

## Data Availability

The raw sequencing data generated in this study have been deposited in the European Nucleotide Archive (ENA) under the project accession number PRJEB74628.

## SUPPLEMENTAL MATERIAL

Supplemental raw data spectrum.02914-24-s0001.pdf The file contains supplementary data related to a study on transcriptomic immune responses during SARS-CoV-2 Omicron B.1.1.529 infection.

ASM does not own the copyrights to Supplemental Material that may be linked to, or accessed through, an article. The authors have granted ASM a non-exclusive, world-wide license to publish the Supplemental Material files. Please contact the corresponding author directly for reuse.
