# Supplementary material for: Decoding the transcriptome from bulk RNA of infection-naïve versus imprinted patients with SARS-CoV-2 Omicron B.1.1.529
Source: Microbiol Spectr. 2025 Jul 9;13(8):e02914-24. doi: 10.1128/spectrum.02914-24 (PMC12323659; doi:10.1128/spectrum.02914-24)
Supplement: Supplemental raw data — The file contains supplementary data related to a study on transcriptomic immune responses during SARS-CoV-2 Omicron B.1.1.529 infection. [file spectrum.02914-24-s0001.pdf]

Appendix

group	Internal no	status	sex	age	severity	Ct T1	BAU/mL	Reads T1	Reads T2	Mean Read Length (bp) T1	Mean Read Length (bp) T2	[illegible]
A	LK21	naïve	m	60	moderate	23	0	6.57E+06	9.73E+06	132	139	
A	LK30	naïve	w	64	mild	31	0	5.64E+06	6.95E+06	138	139	
A	LK31	naïve	w	59	moderate	30	18	6.35E+06	7.82E+06	139	134	
A	LK32	naïve	m	60	moderate	22	0	8.97E+06	7.94E+06	141	133	
A	LK60	naïve	w	78	moderate	39	0	3.14E+06	8.21E+06	122	131	
	mean A			64.20		29	4	6.14E+06	8.13E+06	134.40	135.20	
	sd A			7.95		7	8	2.09E+06	1.01E+06	7.70	3.63	
B	LK34	unvacc_rec	w	36	mild	33	15	1.21E+07	8.26E+06	137	129	
B	LK68	unvacc_rec	w	32	moderate	34	48	8.20E+06	7.85E+06	124	119	
B	LK70	unvacc_rec	m	63	moderate	36	61	8.59E+06	7.83E+06	122	122	
	mean B			43.67		34	41	9.63E+06	7.98E+06	127.67	123.33	
	sd B			16.86		2	24	2.16E+06	2.46E+05	8.14	5.13	
C	LK22	vaccinated	m	70	mild	22	3500	6.92E+06	6.88E+06	132	142	
C	LK24	vaccinated	m	38	moderate	32	3500	6.97E+06	6.97E+06	132	134	
C	LK61	vaccinated	w	82	moderate	23	982	8.23E+06	5.96E+06	125	119	
C	LK69	vaccinated	w	63	moderate	31	1000	6.55E+06	6.39E+06	112	124	
	mean C			63.25		27	2246	7.17E+06	6.55E+06	125.25	129.75	
	sd C			18.57		5	1449	7.32E+05	4.68E+05	9.43	10.28	
	total mean			58.8		30	760	7.35E+06	7.56E+06	129.67	130.42	
	total sd			15.9		6	1333	2.17E+06	1.00E+06	8.73	7.91	

Table S1: Listing of the personal information of the individual patients in this study regarding sex, age, severity of the SARS-CoV-2 infection, Ct value at time point 1 (T1), specific IgG titer at T1, shown as BAU/mL, immune status in relation to SARS-CoV-2, as well as the quality characteristics of the individual bulkRNA samples of the patients after RNAseq at time point T1 (at the latest 1 day after laboratory-confirmed SARS-CoV-2 positive PCR in the nasopharyngeal swab), as well as T2 (three months after the first positive swab).

**WikiPathway**	**group**	**combined score**
Type I interferon induction and signaling during SARS-CoV-2 infection	A	141.4
DDX1 as a regulatory component of the Drosha microprocessor	A	114.6
Homologous recombination	A	90.2
Pathways of nucleic acid metabolism and innate immune sensing	A	90.0
Simplified Depiction of MYD88 Distinct Input-Output Pathway	A	66.6
Biomarkers for pyrimidine metabolism disorders	A	62.5
IL-10 Anti-inflammatory Signaling Pathway	A	59.1
15q11.2 copy number variation syndrome	A	57.3
DNA IR-damage and cellular response via ATR	A	55.9
DNA IR-double strand breaks and cellular response via ATM	A	54.0
Somatic sex determination	B	246.6
Canonical and non-canonical TGF-B signaling	B	190.7
Mitochondrial Gene Expression	B	190.7
miRNA targets in ECM and membrane receptors	B	164.6
Mammalian disorder of sexual development	B	135.6
FGFR3 signaling in chondrocyte proliferation and differentiation	B	114.4
Ectoderm Differentiation	B	103.3
Cholesterol Biosynthesis Pathway	B	52.1
Mevalonate pathway	C	192.4
Cholesterol Biosynthesis with Skeletal Dysplasias	C	115.8
Disorders of the Krebs cycle	C	115.8
Mevalonate arm of cholesterol biosynthesis pathway	C	115.8
Complement and Coagulation Cascades	C	60.4
**Complement Activation**	**AB**	**4054.0**
**Oxidative Damage**	**AB**	**1857.4**
**Allograft Rejection**	**AB**	**1747.0**
**Host-pathogen interaction of human CoV**	**AB**	**989.2**
**Microglia Pathogen Phagocytosis Pathway**	**AB**	**857.4**
**Immune response to tuberculosis**	**AB**	**689.0**
**Allograft Rejection**	**AB**	**586.2**
**Non-genomic actions of 1,25 dihydroxyvitamin D3**	**AB**	**393.4**
**Type II interferon signaling (IFNG)**	**AB**	**363.5**
**COVID-19 adverse outcome pathway**	**AB**	**249.6**
ZNF366	AC	n.a.

Table S2: Listing of all WikiPathways that occur i) group-specifically or ii) overlapping groups. After calculating the differentially expressed genes in the acute phase of an infection with SARS-CoV-2 B.1.1.529, compared with the convalescent phase of the same patient three months later, the patients were divided into the three groups A (infection-naïve), B (imprinted by previous infection) and C (first imprinted by vaccination). In the Venn diagram, the DEGs were sorted into group-specific and cross-group. Subsequently, the filtered DEGs were subjected to an analysis in Enrichr, where the DEGs are assigned to specific biological processes or pathways. In addition, the analysis calculates the p-value, an adjusted p-value, the odds ratio and a combined score of p-value and odds ratio to assess the robustness of the data obtained. Pathways with the highest combined scores (shared DEGs between groups A and B) are highlighted in bold.
